# Demonstration of early functional compromise of bone marrow derived hematopoietic progenitor cells during bovine neonatal pancytopenia through *in vitro* culture of bone marrow biopsies

**DOI:** 10.1186/1756-0500-5-599

**Published:** 2012-10-30

**Authors:** Eleanor Laming, Eleonora Melzi, Sandra FE Scholes, Maira Connelly, Charlotte R Bell, Keith T Ballingall, Mark P Dagleish, Mara S Rocchi, Kim Willoughby

**Affiliations:** 1 Moredun Research Institute, International Research Centre, Pentlands Science Park, Bush Loan, Midlothian, EH26 0PZ, UK; 2 Animal Health and Veterinary Laboratories Agency Lasswade, Midlothian, UK; 3 The Roslin Institute and Royal (Dick) School of Veterinary Studies, University of Edinburgh, Easter Bush, Midlothian, EH25 9RG, Scotland, UK

**Keywords:** Bovine neonatal pancytopaenia, BNP, Colostrum hematopoietic progenitor cells, BM-HPCs, Pluripotential

## Abstract

**Background:**

Bovine neonatal pancytopenia (BNP) is a syndrome characterised by thrombocytopenia associated with marked bone marrow destruction in calves, widely reported since 2007 in several European countries and since 2011 in New Zealand. The disease is epidemiologically associated with the use of an inactivated bovine virus diarrhoea (BVD) vaccine and is currently considered to be caused by absorption of colostral antibody produced by some vaccinated cows (“BNP dams”). Alloantibodies capable of binding to the leukocyte surface have been detected in BNP dams and antibodies recognising bovine MHC class I and β-2-microglobulin have been detected in vaccinated cattle. In this study, calves were challenged with pooled colostrum collected from BNP dams or from non-BNP dams and their bone marrow hematopoietic progenitor cells (HPC) cultured *in vitro* from sternal biopsies taken at 24 hours and 6 days post-challenge.

**Results:**

Clonogenic assay demonstrated that CFU-GEMM (colony forming unit-granulocyte/erythroid/macrophage/megakaryocyte; pluripotential progenitor cell) colony development was compromised from HPCs harvested as early as 24 hour post-challenge. By 6 days post challenge, HPCs harvested from challenged calves failed to develop CFU-E (erythroid) colonies and the development of both CFU-GEMM and CFU-GM (granulocyte/macrophage) was markedly reduced.

**Conclusion:**

This study suggests that the bone marrow pathology and clinical signs associated with BNP are related to an insult which compromises the pluripotential progenitor cell within the first 24 hours of life but that this does not initially include all cell types.

## Background

Bovine neonatal pancytopenia (BNP) is a recently emerged disease of young calves which has been recognised in a number of European countries since 2007 and in New Zealand in 2011 [1-5]. The major clinical signs are associated with thrombocytopenia and include petechiation and external and/or internal haemorrhage. Other haematological abnormalities are described in clinical cases, including leukopenia with both lymphocyte and neutrophil depletion. The underlying lesion is a dramatic bone marrow injury with depletion of hematopoietic cells and their precursors. There is an association with maternal vaccination with an inactivated bovine virus diarrhoea (BVD) vaccine (Pregsure BVD, Pfizer) [6,7]; which was voluntarily withdrawn by the manufacturer in Europe in 2010, but BNP cases continue in calves born to or ingesting colostrum derived from some vaccinated animals. The syndrome can be reproduced by feeding of colostrum from cows which have borne affected calves (BNP dams) to unrelated calves [8-10], and disease can be prevented by colostrum substitution [11]. While individual colostra have a more variable outcome in terms of development of BNP [8,9], the use of pooled colostrum from a number of BNP dams produces a more reliable outcome with a high proportion (80-100%) of calves developing BNP or its typical clinicopathological features [10].

Antibodies present in serum of BNP dams have been shown to be capable of binding to peripheral leukocytes and to cells in bone marrow smears of unrelated normal calves [12] and were detected bound to leukocytes of affected calves [9,13]. Such studies support the hypothesis that maternally derived colostral alloantibody targeting a bovine antigen (or antigens) causes the bone marrow pathology in calves. While the identity of the target antigen(s) is unconfirmed, evidence supporting the presence of anti-bovine MHC Class I antibodies in BNP dams [14] and induction of anti-bovine MHC Class I antibodies by the implicated vaccine has recently been described [15].

While no linkage to MHC-class II nor factor XI has been detected in affected calves there is some evidence that within herds, specific groups or lines of animals may be more frequently affected, suggesting that some cows are more at risk of BNP-associated alloantibody production due to either intrinsic factors or to interaction between the pregnant cow and its fetus [16,17].

The lack of an animal model, reliance on clinical cases and the requirement to use colostral challenge of calves to investigate the disease has severely limited the opportunities for systematic investigation. We describe here a pilot study on the utility of bone marrow hematopoietic progenitor cell (BM-HPC) cultures to assess the functional compromise of harvested bovine BM-HPCs at 24 hours and 6 days post challenge with either pooled BNP dam colostrum or pooled normal colostrum.

## Methods

### Animals

Four male Holstein Friesian colostrum deprived calves were sourced over a period of 1 week from a single dairy farm and fed either challenge (n=2; calves W and Y) or control (n=2; calves X and Z) colostrum pools. To ensure that the calves represented a broad range of MHC genotypes, each calf was genotyped at the highly polymorphic MHC class II *DRB3*[16]. The MHC genotypes of each calf were as follows; W, DRB3*1001, DRB3*1201; X, DRB3*1001, DRB3*1501; Y, DRB3*0101, DRB3*1001 and Z, DRB3*1201, DRB3*1201.

### Colostrum

The challenge pool consisted of a standardised colostrum pool collected from 9 previously identified BNP dams. Challenge calves were fed 2.15 litres of this colostrum pool within 6 hours of birth. Control calves were fed a similarly constituted pool collected from 9 dams sourced from a BVDV free, BVDV unvaccinated farm where no cases of BNP had been observed. All calves were bottle fed colostrum to aid closure of the rumenoreticular groove and optimise antibody absorption. In a previous study we confirmed that this challenge pool induces BNP (data not shown; submitted for publication).

### Clinical scoring

A defined clinical scoring system was used to determine the end point of the experiment. Parameters measured included demeanour, rectal temperature, heart and respiratory rate, presence and duration of petechiation or other abnormal bleeding, packed cell volume (PCV, by microhematocrit) and manual thrombocyte count based on inspection of a stained blood smear (Reastain Quick Diff, Reagena), determination of the average number of platelets in 5 oil immersion fields (100×) and multiplication to generate an estimated thrombocyte count expressed as ×10^9^/litre. All experimental protocols involving animals were approved by the Moredun Research Institute Animal Experiments & Ethical Review Committee and authorised under the UK Animals (Scientific Procedures) Act 1986.

### Analysis of blood samples

Blood samples were taken by jugular venepuncture using a needle and syringe to avoid artefactual thrombocyte clumping. Samples were taken pre-challenge, then at 4,8,12 and 24 hours post-colostrum feeding and daily thereafter. Analysis of colostrum deprivation and subsequent absorption of colostrum was assessed by determination of serum gamma glutamyltransferase (γGT) as a passive marker. Routine haematology was performed (including erythrocyte, thrombocyte and total leukocyte counts, MVC, MCHC and white blood cell differential count) at a commercial laboratory (SAC Veterinary Services, Penicuik).

Quantification of lymphocyte subset depletion was performed using an indirect fluorescence staining and flow cytometric analysis as previously described [18]. Erythrocytes were lysed using an ammonium-chloride based reagent [19] and leukocytes washed and resuspended in FACS buffer (PBS, 5% heath inactivated FBS, 0.02%NaN_3_) for the labelling with the following antibodies: IgG1 and IgG2a isotype controls (Moredun, UK, 2 μg/ml), anti bovine CD3 (VMRD, USA, 1 μg/ml), CD4 (VMRD, USA, 2 μg/ml), CD8 (ILRI, Kenya, 1 μg/ml) and γ/δ TCR (VMRD, USA, 1.25 μg/ml), anti bovine NK p46 (Serotec, UK, 1 μg/ml), anti bovine CD25 (VMRD, USA, 1 μg/ml), anti bovine CD21 (Serotec, UK, 1 μg/ml ), and anti bovine SIRP-α (monocytes) (ILRI, Kenya, 1 μg/ml). Labelling was detected with goat-anti mouse IgG Alexa 488 conjugate (Invitrogen UK, 1 μg/ml). Up to 20,000 events/ per samples were acquired with a CyAn ADP flow cytometer (Beckman Coulter, USA) and analysed with Summit software (Dako, USA).

### Bone marrow biopsy

We performed extensive assessment of the preferred site (femur or sternum) and method (core biopsy or aspirate) for bone marrow sampling using similarly aged calves euthanased for other reasons. We found that the most reliable site of bone marrow biopsy was the sternum, and that a core biopsy was the most reliable and reproducible method for both histopathological examination and for collection of undamaged, viable cells in the numbers required for the clonogenic assay described below. Evidence that approach used was effective included good reliable harvests of viable cells for culture and no evidence of cell damage or artefactual depletion in the histological preparations. Sternal bone marrow biopsies were taken under reversible sedation (intravenous 10 μg/kg medetomidine hydrochloride, reversed with atipamazole) and local analgesia (20 mg lignocaine hydrochloride with adrenaline and 5 mg of bupivicaine hydrochloride) at 24 hours and 6 days post challenge using a 0.5 cm diameter trephine needle (Rocket Medical, Washington, UK). The animals were closely monitored after biopsy for signs of discomfort, pain, bleeding or infection; none were detected.

### Clonogenic assay of hematopoietic progenitor cells

A protocol based on a previously described method [20] was followed with some minor modifications. Briefly, following the biopsy procedure, the bone marrow core was released from the needle and placed immediately into a universal container with 6ml of collection medium (CM: Iscove’s Modified Dulbecco’s Medium (IMDM) supplemented with 10% heat inactivated FBS, 8 mM L-glutamine, 100 IU penicillin/100 μg streptomycin per ml, 1.25 μg/ml of amphotericin B and 20 IU/ml of lithium heparin) and placed on a rotary suspension mixer for 30 minutes at room temperature (RT). The released cell suspension was harvested and the residual biopsy core fixed in formalin and, following decalcification, routinely processed for histological examination (see below). The cell suspension was layered onto 6 ml of Lymphoprep (Axis-Shield, UK) density gradient separation medium (density 1.077 g/ml) and centrifuged at 1175 g for 30 minutes at 15°C. The interface (mononuclear cell layer), was collected and washed 3 times with washing medium (WM: as CM above but without heparin), with centrifugation at 250 g for 10 minutes at 4°C between each wash. After the final wash, the cell pellet was resuspended in 1ml WM.

A viable cell count was performed on the final cell suspension using 0.2% nigrosin and a haemocytometer. The concentration was adjusted to 1 × 10^6^ live cells per ml in WM. The cell suspension was then diluted 1/10 in a commercial methylcellulose culture medium containing human recombinant erythropoietin (Methocult® H4034 Optimum with EPO, Stemcell Technologies, UK) to achieve a final concentration of 1 × 10^5^ cells per ml. Granulocyte/macrophage colony-stimulating factor (rboGM-CSF expressed in CHO cells using a previously defined protocol for recombinant cytokine expression [21], a gift from Prof. G. Entrican, Moredun) was added to the suspension at the previously determined optimum dilution for DC culture, according to the method of Werling et al. [22]. The media and cells were vortex mixed thoroughly and left to stand for 10 minutes to allow air bubbles to dissipate prior to plating. Each sample was plated in duplicate, adding 1.1 ml to each of two 35 mm culture dishes using 3 ml syringes and 16-gauge blunt-ended needles (Stemcell technologies, UK). On one occasion (calf Y day 6) insufficient cell numbers were obtained for 2 dishes and a single dish was prepared. Quadrants were marked out and numbered on the underside of each dish to facilitate colony counting. The two dishes were placed in a 100 mm culture dish with a third uncovered dish containing sterile distilled water, and placed in a humidified incubator at 37°C with 5% CO_2_.

### Colony scoring

Prior to the study, the method for identifying colonies was developed using bone marrow biopsy material opportunistically obtained from calves euthanased for other reasons. Preliminary colony identification was made through assessment of morphological characteristics of the colony itself and of the single cells of which it was composed. This assessment was confirmed by picking up the colony and examination of a stained smear and/or cytospin preparation.

For the study samples, microscopic examination of the cultures was performed using a 60 mm gridded scoring dish as a template to facilitate counting. Cell colonies were counted and typed as CFU-GEMM, CFU-E and CFU-GM. As per previous studies [23-25] clusters of cells were considered colonies for CFU-GM when more than 40 cells were counted at day 7 and for CFU-E when more than 8 haemoglobinised cells were counted at day 5. CFU-GEMM are considered multilineage colonies with the largest number of cells but are not previously characterised in cattle; we counted colonies of greater than 10 cells at day 2 and greater than 100 cells at day 7.

## Results

### Hematology

**Figure 1:**
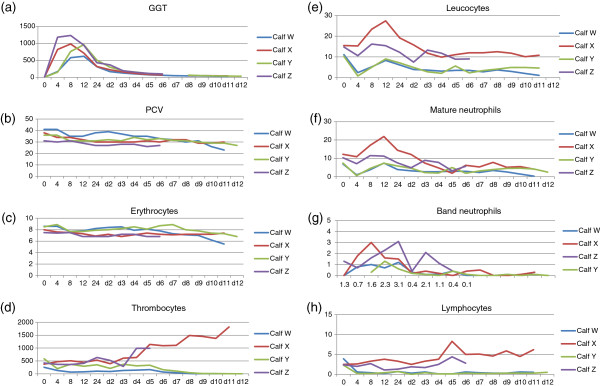
**Results of biochemical and haematological testing.** The scale on the X- axis for all graphs is in hours for time points 0,4,8,12 and 24 and for days 2–12 thereafter. The scale on the Y-axis is as appropriate for the parameter measured (see appropriate sub-legend). **(a)** results of testing for GGT (iu/litre) to demonstrate adequate colostral absorption. **(b)** PCV (%): no difference was observed between challenge (X,Z) and control (W,Y) calves. **(c)** Erythrocyte count (x10^12^/litre): no difference was observed between challenge (X,Z) and control (W,Y) calves. **(d)** Thrombocyte count (x10^9^/litre): challenge calves (W and Y) demonstrate a marked reduction in thrombocyte counts when compared to controls (X and Z). **(e)** Leukocyte count (x10^9^/litre): challenge calves (W and Y) demonstrate a marked reduction in leukocyte counts when compared to controls (X and Z). **(f)** Mature neutrophil count (x10^9^/litre): no difference was observed between challenge (X,Z) and control (W,Y) calves. **(g)** Band (immature) neutrophil count (x10^9^/litre): no difference was observed between challenge (X,Z) and control (W,Y) calves. **(h)** Lymphocyte count (x10^9^/litre): challenge calves (W and Y) demonstrate a marked reduction in lymphocyte counts when compared to controls (X and Z). Note: These results have no level of statistical significance indicated as there are only 2 animals in each group. Values are only available until day 6 for calf Z which was euthanased for welfare reasons unrelated to the experiment.

Calves which received challenge colostrum developed marked thrombocytopenia by day 6 and marked lymphopenia between 4 and 8 hours post-challenge (Figure 1), consistent with the pre-clinical stages of BNP. These alterations persisted until euthanasia. No abnormalities were observed in the control animals.

### Peripheral lymphocyte depletion

**Figure 2:**
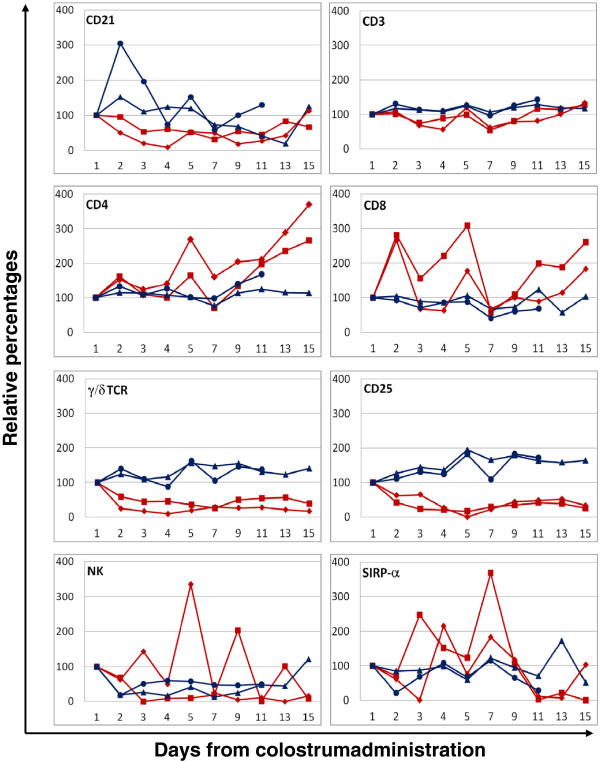
**Changes in the relative percentages of peripheral blood mononuclear cells following control or challenge colostrum administration.** Relative percentages were calculated as increase/decrease in the proportion of cells normalised to the pre-administration values (100%). Time points (X axis) were as follow: 1: pre-challenge; 2: 4 hours post challenge; 3: 8 hours; 4: 12 hours; 5: 24 hours; 7: day 3; 9: day 5; 11: day 7; 13: day 9 and 15: day 11. Animal Z was humanely euthanized at time point 11. Blue lines: control animals (triangle calf X, diamond calf Z); red lines: challenged animals (square calf W, triangle calf Y).

Blood samples collected from the two challenged and two control animals were typed for specific lymphocyte subsets and the number of population-specific cells displayed as percentage increase or decrease from the initial pre-challenge values (Figure 2). For all but the CD21+ve subpopulation (B cells) the data obtained in the unchallenged animals was remarkably consistent and showed only minor changes over time. The challenged animals displayed a larger variability in their subpopulation values however, and a clear reduction in the proportion of CD25^+ve^, CD21^+ve^ and γ/δ TCR^+ve^ cells was observed immediately after challenge. CD3, CD4 and CD8^+ve^ cells did not show a similar decline; on the contrary we noticed an increase in CD4^+ve^ and CD8^+ve^ relative percentages towards the later stages of the observation period. The values of NK cells and SIRP-α positive cells (monocytes) were too variable to identify specific trends.

### Clinical and post mortem findings

**Figure 3:**
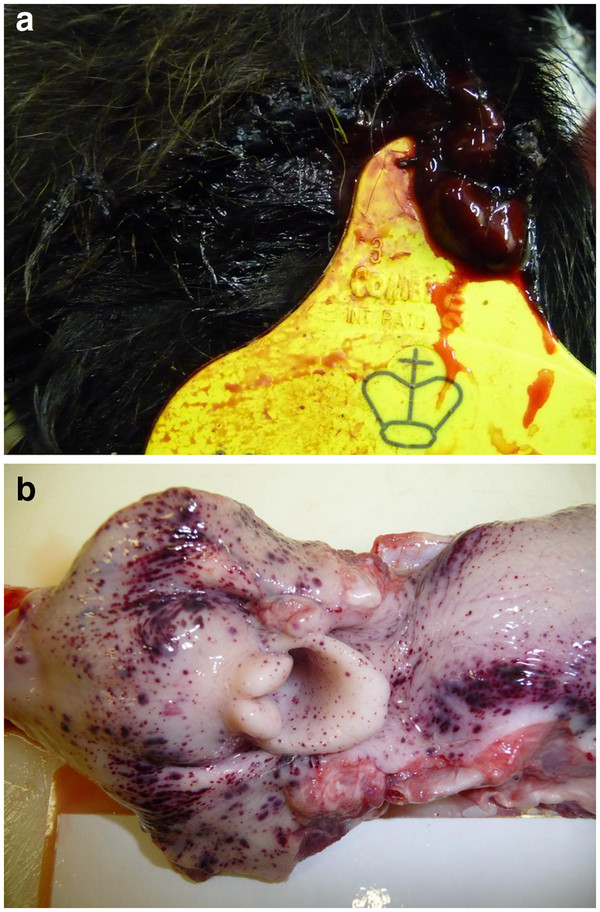
**a: Haemorrhage from the ear tag of challenged calf Y 12 days post challenge. b**: Petechiation of the laryngeal and pharyngeal tissues at necropsy.

**Figure 4:**
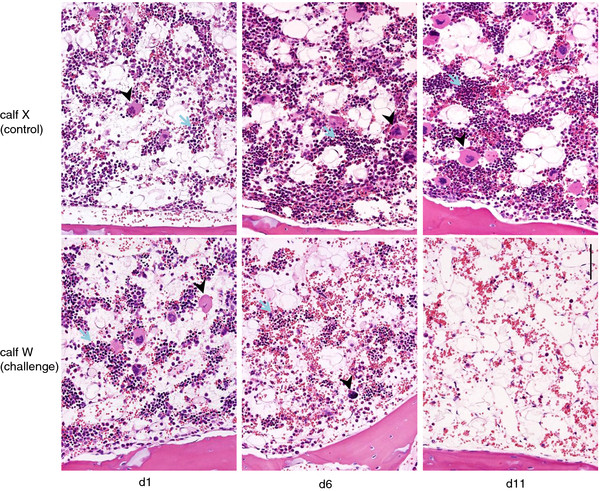
**Sequential bone marrow histology of control calf X (upper row) and challenge calf W (lower row) at days 1, 6 and 11. Examples of erythroid clusters are indicated by blue arrows and examples of megakaryocytes by black arrowheads.** Left column: Biopsy cores at day 1 from challenge calf (W) and control calf (X) contain similar distributions of erythroid clusters and megakaryocytes. Centre column: By day 6, the numbers of haematopoietic cells (HPC) including megakaryocytes has markedly decreased in the challenge calf, whereas there is an increased density of HPC in the control calf compared with the earlier time point. Erythroid clusters in the challenge calf are composed mainly of cells in the later stages of differentiation. Right column: At necropsy on day 11, trilineage hypoplasia with absence of all HPC clusters is found in the challenge calf whereas there is no change in the control compared with day 6: Bar = 100 μm (applies to all images).

Petechiation and bleeding from the ear tag was observed in both challenged calves from day 7 (Figure 3a) and euthanasia was performed in accordance with the clinical scoring system at day 11 (calf W) and 12 (calf Y). Post-mortem examination (PME) demonstrated gross lesions consistent with BNP (Figure 3b) and histopathological examination of bone marrow confirmed both challenged calves had lesions of trilineage hypoplasia at the time of euthanasia (Figure 4). The control calves were not clinically affected, did not develop any haematological abnormalities, bone marrow lesions, nor were gross or histological lesions observed at necropsy. These findings confirmed the induction of BNP in both challenge calves.

### Histopathology: sternal bone marrow biopsy

All sternal biopsy cores contained substantial amounts of medullary intertrabecular tissue. No difference was detectable between the challenged calves and the control calves at day 1, with moderately abundant myeloid and erythroid series cells and megakaryocytes at varying stages of maturation (Figure 4). In contrast, in the challenge calves at day 6 there were marked reductions in the numbers of megakaryocytes, which were mostly mature (stage 3), and the myeloid and erythroid series cells were mainly represented by mature neutrophils and eosinophils and by late normoblasts (Figure 4), whereas the haematopoietic cell density had increased in both control calves compared with day 1.

### Hematopoietic progenitor cell cultures

**Figure 5:**
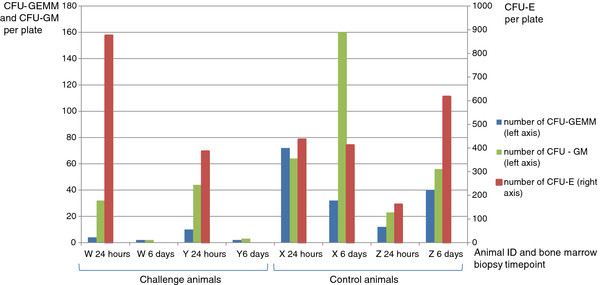
**Colony counts in BM-HPC cultures from biopsies taken at 24 hours and 6 days post-colostral challenge.** Calves W and Y are challenge animals, calves X and Z are controls. At 24 hours, no difference is observed between animals/groups in terms of colony counts, though morphological differences were observed (see text and figure 3). At 6 days post-colostral challenge all three lineages are markedly reduced in both challenged calves (W and Y) as compared to the control animals (X and Z). No CFU-E were observed in either challenged animal at 6 days.

BM-HPC cultures demonstrated normal colony development in the control animals at both sampling times (based on previous observations of cultures from healthy calves during protocol development and validation). The low number of animals used and variation between colony counts precludes statistical analysis of cloning efficiencies for the 24 hour biopsy, however, morphological assessment was possible (see below). By day 6 marked differences were observed between the control and challenge groups in terms of colony counts for all three colony types (Figure 5).

**Figure 6:**
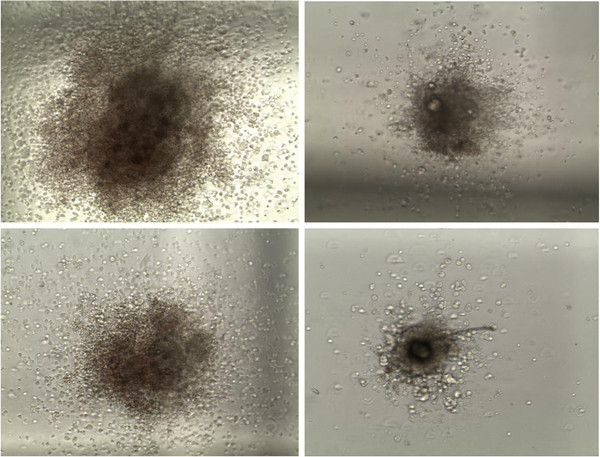
Typical CFU-GEMM colonies identified on day 7 of culture in control (left) and challenge (right) calves for 24h (top row) and 6 day (bottom row) post challenge biopsies (x10).

At the 24 hour biopsies, in both control and challenge animals, CFU-E colonies were clearly visible with a distinct reddish colour due to hemoglobinisation and were of similar size and morphology. However for the day 6 biopsies, while CFU-E colonies developed normally in the control animals, no CFU-E colonies were identified in the challenge animals. Similarly, for CFU-GM, colony number and morphology were preserved at 24 hours for both control and challenge animals. However by day 6, the number of CFU-GM was markedly reduced in samples from challenge animals as compared to the controls (Figure 5).

There was a marked difference between the two groups with respect to size and morphology of the CFU-GEMM colonies, in both the 24 hour and 6 day samples (Figure 6). Colonies from control calves’ biopsies were composed of a higher number of cells, distributed over a wider area around the compact centre of the colony, while colonies from challenge calves’ biopsies were composed of fewer, larger cells and had more compact centres.

## Discussion

This is the first etiopathogenesis study of BNP which assesses the functionality of BM-HPCs. The results shown here demonstrate that BNP was induced in the challenge animals and that functional damage to the hematopoietic progenitor cells was apparent prior to the development of clinical signs, gross or histopathological lesions. As early as 24 hours after colostrum intake, the CFUs-GEMM were compromised in their colony forming ability and by day 6 the number of all CFU types was markedly reduced. This supports the hypothesis that the main target cell is the pluripotent hematopoietic progenitor cell. In addition, this study further demonstrates that the more differentiated cells (CFU-E and CFU-GM precursors) present in the bone marrow are apparently not compromised at 24 hours post-colostrum ingestion.

Lymphopenia post-colostral challenge has been previously observed [9,10,12]; therefore in this study we attempted to determine whether a specific subset of peripheral blood mononuclear cells (PBMC) was depleted. We failed to demonstrate any subset-specific depletion, but did observe a tendency for CD25^+ve^, γδT cell and B cell percentages to drop and subsequently fail to rise when compared to control animals. B-cell lymphopoesis begins in the fetal liver with transition to the bone marrow shortly after birth [26]. The observed failure of B-cell percentages to rise after colostrum ingestion suggests that B lymphocytes may be removed from the circulation during this transition stage. γδT cells are enriched at epithelial surfaces as well as being the pre-eminent blood lymphocytes subpopulation in young ruminants [27]; migration of γδT-cells from the thymus toward peripheral tissues increases markedly during fetal life and after birth [28]. In analogy with B cells, it is possible that these cells are also removed from the circulation at this critical homing stage. CD25^+ve^ cells represent activated or regulatory B and T cells, as well as some thymocytes and myeloid precursors (such as those which coexpress CD13, CD33, CD34, MY8, and HLA-DR, and lack CD14 or CD11b being at or near the myeloblast stage of differentiation [29]). CD25 is also transiently expressed at a low level during normal B-cell development (pre-B cell stage in the bone marrow [30] and at a higher level during a very early stage of T-cell development in fetal and adult thymus (double negative stage) [31]. This very early removal of lymphocytes from the peripheral circulation, taken in association with the previous demonstration of binding of alloantibodies [8,14,15] support the hypothesis that one or more common antigen(s) present on bone marrow HPCs and lymphocytes are targeted almost immediately after colostrum absorption.

Two groups have recently published evidence demonstrating the presence of -MHC-class I specific antibodies on circulating leukocytes of affected calves [14,15]. While the induction of such antibodies during BNP is clear, specificity for the broadly expressed classical MHC-I molecules may not be responsible for the disease syndrome *per se* as they would be expected to target cells more widely than the haematopoietic lineages (bone marrow and peripheral lymphocytes). Our observations suggest that alloantibodies targeting other common antigens could be responsible for the bone marrow lesion, due to the apparent sparing effect on CFU-E and CFU-GM at the 24 hour biopsy as well as peripheral granulocytes throughout the clinical progression. Expression of MHC-I is widespread and includes both CFU-E and CFU-GM in cattle [23,32] but the presence of MHC-I on bovine CFU-GEMM, while it would be expected, has not been confirmed. Possible explanations could include a difference in the levels of MHC-I expression in the highly active CFU-GEMM, a difference in their susceptibility to damage, or a difference in cell-type specific expression of an unusual non-classical MHC- I specificity which could make these cells more sensitive to antibody-dependent damage (either via ADCC or complement-mediated). Further studies in this area are required to clarify the exact mechanism(s).

## Conclusion

This study demonstrates the utility of *in vitro* BM-HPC culture in the investigation of BNP. This may facilitate further studies of early pathogenesis, which is not possible by investigation of natural cases, since once the clinical features are apparent the bone marrow lesion is already too advanced to define its pathogenesis.

We show that pluripotent CFU-GEMMs are functionally compromised within the first 24 hours post colostrum ingestion and that all colony types examined are damaged by day 6. This method will facilitate *in vitro* studies to further characterise the aetiology, maternal vaccinal responses, colostral antibody titre and specificity in a standardised, non-animal model system. In addition we show that the profound lymphopenia observed in the early stages of BNP does not appear to be subset- specific and the differences observed in the behaviour of B and γδ T cells are probably related to the inability of the thymus to respond to environmental pressures by increasing cellular output.

Further development of such *in vitro* cell culture systems should improve opportunities to investigate the functional target of the alloantibodies in BM-HPCs in BNP and could allow investigation of the potential risk to other species, including man, of consumption of colostrum or milk from affected cows as well as assessment of the safety of inactivated vaccines in pregnancy.

## Competing interests

The authors have no competing interests in this study.

## Authors’ contributions

MR and KW conceived the study, performed clinical and laboratory work and contributed to and critically reviewed the manuscript. EL and EM developed the BM-HSC methodologies, optimised and performed the *in vitro* cultures and drafted the manuscript. KTB performed and interpreted the MHC-II genotyping. MD performed post-mortem examinations and MD and SFES performed histopathological interpretation of biopsy and post mortem material. MC acquired and analysed the FACS samples. CRB supplied the colostrum and provided the methods for the bone marrow biopsy procedure and for clinical scoring. All authors read and approved the final manuscript.
